# Genomic and Phenotypic Safety Assessment of Probiotic *Bacillus coagulans* Strain JBI-YZ6.3

**DOI:** 10.1007/s12602-024-10305-4

**Published:** 2024-06-19

**Authors:** Yongmei Zhang, Tom J. Overbeck, Victoria L. Palmer Skebba, Neil N. Gandhi

**Affiliations:** Jeneil Biotech, Inc, Saukville, WI 53080 USA

**Keywords:** *Bacillus coagulans*, Probiotics, Safety assessment, Genomic analysis, Antibiotic resistance, Phylogeny

## Abstract

Spore-forming *Bacillus coagulans* has been widely recognized as an important probiotic, which is commonly used in products for human consumption and animal feeds. *B*. *coagulans* exhibits beneficial traits from both *Bacillus* and lactic acid-producing *Lactobacillus*. The present study evaluated the safety of the newly isolated *B. coagulans* strain JBI-YZ6.3, using combined genomic and phenotypic analysis approaches. The taxonomic classification based on genome sequence and biochemical tests identified strain JBI-YZ6.3 as *B. coagulans*. Comprehensive genome-based analyses established JBI-YZ6.3 as a novel strain of *B. coagulans*. Antibiotic susceptibility testing showed that the strain JBI-YZ6.3 was sensitive to a panel of fourteen antibiotics, and no genes related to antibiotic resistance were found in its genome. The spores of strain JBI-YZ6.3 exhibited tolerance to acid and bile salts, as well as stability at ambient and elevated conditions of temperature and relative humidity. There were no homologs of *Bacillus* toxin genes identified in the genome of JBI-YZ6.3, and the strain exhibited no cytotoxicity towards Vero cells and human peripheral blood mononuclear cells. In conclusion, findings from this study support the safety of *B. coagulans* strain JBI-YZ6.3, which can be developed into new probiotic products for preventive and therapeutic benefits in human and animal hosts.

**Supplementary Information:**

The online version contains supplementary material available at 10.1007/s12602-024-10305-4.

## Introduction

Probiotics are live microorganisms that, when consumed in adequate quantity, provide health benefits to the host [1]. Probiotic microorganisms, including bacteria and yeasts, are naturally present in various fermented foods, such as yogurt, natto, kimchi, and sauerkraut. Probiotic organisms can also be added to other food products and administered as dietary supplements to provide health advantages.

The most well-accepted benefits of probiotics are to support a healthy gut microbiota, the digestive tract, and the immune system. Probiotics produce bacteriocins and short-chain fatty acids (SCFAs) that inhibit pathogens, creating a more favorable gut environment for beneficial commensals and a balanced microbiome [2, 3]. Probiotics promote digestive health by improving barrier functions and producing digestive enzymes to metabolize nutrients [4, 5]. Probiotics also possess immune-modulating and anti-inflammatory properties via their effects on both innate and adaptive immunity. Diverse clinical studies have demonstrated the efficacy of probiotics in the treatment and prevention of gastrointestinal diseases such as antibiotic-associated diarrhea, infectious diarrhea, inflammatory bowel syndrome (IBS), ulcerative colitis, and abdominal pain and bloating [6–9]. Further value of probiotics has also been demonstrated in treating urinary tract infections, immune disorders, cancer, lactose intolerance, and allergies [10, 11].

The most well-known probiotic bacteria are lactic acid-producing *Lactobacillus* and *Bifidobacterium* organisms. Spore-forming *Bacillus* probiotics have attracted more attention in recent years due to their intrinsic ability to survive harsh industrial processing conditions (thermal processing and low moisture) and passage through the gastrointestinal tract (low pH and bile salts) [12, 13]. Specifically, *Bacillus coagulans* exhibits characteristics of both *Bacillus* and *Lactobacillus* and has been developed into various commercial products (BC30, LactoSpore, Sporlac, LACBON). The probiotic benefits of *B. coagulans* have been extensively studied and well documented [14–16]. In 2020, *B. coagulans* was taxonomically reclassified to *Weizmannia coagulans* based on comparative genomics analysis [17]. Because *B. coagulans* is how this species has been recognized in a multitude of publications, this nomenclature is widely recognized by consumers and regulatory authorities; consequently, *B. coagulans* will be used as the species name in this report.

We have recently isolated a new *B. coagulans* strain JBI-YZ6.3 from tapioca starch and sequenced its genome [18]. Here, we report a comprehensive study on the stability and safety of strain JBI-YZ6.3 using integrated genomic- and phenotypic-based approaches. Genome-based analysis tools were used to compare strain JBI-YZ6.3 with known *B. coagulans* strains. Phenotypes of JBI-YZ6.3 associated with its safety were correlated to its genotypic properties. These findings provide the basis for strain JBI-YZ6.3’s safety, a prerequisite for the strain to be further developed into new probiotic products.

## Methods

### Strain and Growth Conditions

*B. coagulans* strain JBI-YZ6.3 was isolated from tapioca starch [18]. The strain was deposited at the American Type Culture Collection (ATCC) as PTA-127366. Strain JBI-YZ6.3 was routinely maintained on glucose-yeast extract (GYE, recipe see “*Bacillus coagulans* GBI-30, 6086”, FCC Monographs) medium at 40 °C under aerobic conditions.

### Phylogenetic Analysis by 16S rRNA Sequencing and MLST

The primers used for 16S rRNA amplification and sequencing were 8F (5′-AGAGTTTGATCCTGGCTCAG) and 1541R (5′-AAGGAGGTGATCCANCCRCA) [19]. The NCBI standard BLASTN was used to search for sequences with significant similarities in the type-strain 16S rRNA database. The phylogenetic distance of strain JBI-YZ6.3 with selected *Bacillus*-type strains inferred with the fast minimum evolution (FastME) method was calculated from pairwise alignments and displayed using the Molecular Evolutionary Genetics Analysis (MEGA v. 10.0.4) program. Multi-locus-sequence typing (MLST) analysis of strain JBI-YZ6.3 was done by using five housekeeping gene fragments (*gyrB*, *ilvD*, *ldh*, *pta*, and *rpoB*). The same primers were used for high-fidelity PCR and sequencing (Supplementary Table S1). The NCBI standard BLASTN was used to search for sequences with significant similarities to the concatenated sequence of strain JBI-YZ6.3 from the five MLST gene fragments. Clustal Omega (v 1.2.4) was used to generate a multiple sequence alignment of strain JBI-YZ6.3’s sequence with its homologous sequences from twenty different *B. coagulans* strains. Phylogenetic distance inferred with the neighbor-joining clustering method was calculated and displayed using the MEGA program.

### Identification by Carbohydrate Metabolism Profiling

BioMérieux API 50 CHB strips were used to determine the ability of strain JBI-YZ6.3 to metabolize carbohydrates. Colonies of strain JBI-YZ6.3 from a GYE agar plate were used to prepare the inoculum. The assay was performed according to the manufacturer’s instructions and was incubated at 30 °C. Results were recorded after 24 h and 48 h incubation and analyzed using APIWEB™ to establish an identification.

### Comparative Genomic Analyses

The genome of strain JBI-YZ6.3 was sequenced using the long-read PacBio platform [18]. The genome sequence of JBI-YZ6.3 was annotated using the RASTtk-enabled Genome Annotation Service [20]. Eight fully assembled genomes of *B. coagulans* that are publicly available were used in three different genome-based comparative analyses. The Genome-to-Genome Distance Calculator (GGDC) 3.0 (https://ggdc.dsmz.de/ggdc.php) was used to calculate the genome-based digital DNA-DNA hybridization (DDH) values. The average nucleotide identity (ANI) was estimated using the ANI calculator (http://enve-omics.ce.gatech.edu/ani/), which estimates the average nucleotide identity using both best hits (one-way ANI) and reciprocal best hits (two-way ANI) between two genomic datasets [21]. The Similar Genome Finder service of BV-BRC (https://www.bv-brc.org/app/GenomeDistance) was used to compute genome distance and k-mer counts.

### Shelf-Life Stability

Freeze-dried JBI-YZ6.3 spores were blended with maltodextrin to achieve > 15 billion CFU/gram. Samples from three production lots (2 g) were aliquoted into Whirl–Pak bags and heat sealed in aluminum bags. The sample bags were stored under two different conditions: one at 40 °C and 75% relative humidity and the other at room temperature (average 25 °C) and ambient humidity (30–60%). At each sampling time point, one bag of each lot was used to enumerate viable spores by serial dilutions and pour plate method. One gram of JBI-YZ6.3 spore sample was mixed with 99 mL G-saline (0.85% NaCl and 18.75 mg/L sodium lauryl sulfate) in a glass bottle and hydrated at room temperature for 30 min. The suspension was sonicated to disperse cell clumps (Sonicator 3000 with Microtip settings: amplitude = 38; process time = 5 min with 20 s on and 10 s off). The sonicated sample was heat-treated at 80 °C for 20 min and serially diluted by 10 folds in G-saline. One milliliter of diluted samples was transferred to a petri dish and mixed with 15 mL GYE agar (pre-warmed at 50 °C). After the agar solidified, the plates were incubated at 40 °C for 2 days before colony counting. Dilutions at 10^−8^ and 10^−9^ were used for colony counting as these two dilutions produced between 30 and 300 colonies on each plate. Each dilution was counted in triplicate, and an average CFU/mL was estimated.

### Acid and Bile Tolerance

Spores of strain JBI-YZ6.3 were suspended in phosphate-buffered saline (PBS) adjusted to acidic pH 2, 3, and 4 at a cell density of 1 × 10^9^ cells/mL. After incubation at 37 °C for 1, 2, and 3 h, an aliquot of the treated sample was removed to enumerate viable cells by serial dilutions and pour plate method as described above. To determine tolerance to bile, JBI-YZ6.3 spores were suspended in G-saline, G-saline with 0.3% bovine bile, or G-saline with 0.5% bovine bile and incubated at 37 °C. After incubation for 1, 2, 3, and 4 h, an aliquot of the treated sample was removed to enumerate viable cells. The data from acid and bile tolerance assays were analyzed using a two-way ANOVA test.

## Antibiotic Susceptibility Testing (AST)

The minimal inhibitory concentrations (MICs) of a panel of antibiotics against strain JBI-YZ6.3 were determined by the Epsilometer test (E-test) using MIC test strips (Liofilchem). Colonies of strain JBI-YZ6.3 on GYE agar were suspended in saline to reach a cell density matching the 0.5 McFarland standard. Inoculation on GYE agar and MIC test strip applications was done according to the manufacturer’s procedures. The plates were incubated at 40 °C for 20 h. The MIC values were determined by reading where inhibition ellipses intersected the scale.

## Detection of Enterotoxins and Cytotoxicity

The *Bacillus cereus* enterotoxin-reversed passive latex agglutination (BCET-RPLA) toxin detection kit was used to test the cell-free supernatant of strain JBI-YZ6.3. The cytotoxicity assay was adapted from the guidance provided by the European Food Safety Authority (EFSA), and the release of lactate dehydrogenase (LDH) was used as a readout for cell death [22]. Vero cells (ATCC CCL-81) in 24-well plates were covered in extracellular bathing (EC) solution for 10 min to equilibrate and then treated with the cell-free supernatant of JBI-YZ6.3 to a concentration of 10% (v/v). At 60 min, LDH released into the EC buffer was quantitated using a LDH detection kit (Promega). All test conditions were conducted in triplicate. Baseline LDH was established by assaying untreated Vero cells. The maximum LDH content was determined by lysis of cell monolayer by the addition of Lysis Solution (Promega).

## Viability of Peripheral Blood Mononuclear Cells in the Presence of JBI-YZ6.3

The cytotoxicity of strain JBI-YZ6.3 was also measured against peripheral blood mononuclear cells (PBMC) using germinated spores. Dry spores of JBI-YZ6.3 were suspended in sterile PBS (40 mg/mL), hydrated, sonicated, and heat-treated as described above. The suspension was cooled immediately to 45 °C with intermittent vigorous shaking and diluted tenfold to create a stock solution, which was used to prepare serial dilutions to treat PBMC cultures at 0.4, 0.2, 0.1, 0.05, 0.025, 0.013, 0.006 mg/mL.

PBMC were purified as described previously [23]. Briefly, peripheral venous blood was drawn from three human healthy donors upon written informed consent, as approved by the Sky Lakes Medical Center Institutional Review Board (FWA 2603). The blood was drawn into heparin vacutainer vials, and the PBMC were isolated using Lympholyte Poly (Cedarlane) by centrifugation at 400 × g for 35 min. The PBMC were washed twice in PBS, counted, and adjusted to a cell density of 10^6^/mL using RPMI 1640 medium containing 10% heat-inactivated fetal calf serum and 100 U/mL penicillin–100 mg/mL streptomycin.

Serial dilutions of germinated JBI-YZ6.3 spores were added to PBMC cultures in U-bottom 96-well cell culture plates at a density of 10^6^ cells/mL in a volume of 0.2 mL. Each dose of germinated JBI-YZ6.3 spores was tested in triplicate, and untreated control cultures were tested in hexaplicate. After incubation at 37 °C for 20 h, cell survival was determined based on live-versus-dead staining using flow cytometry and mitochondrial metabolic activity using the 3-(4,5-dimethylthiazol-2-yl)-2,5-diphenyltetrazolium bromide (MTT) assay.

For the flow cytometric cell viability assay, the PBMC cultures were transferred to V-bottom 96-well microtiter plates, washed once in PBS, and analyzed by an Attune flow cytometer and software (Thermo-Fisher Scientific), using the forward and side scatter plots to gate on live versus apoptotic lymphocytes. The commercial kit CyQUANT MTT Cell Viability Assay Kit (Thermo-Fisher Scientific) was used in the MTT assay. The MTT reagent was added to the PBMC cultures at a final concentration of 12 mM and incubated for 4 h to allow the conversion of MTT to formazan by mitochondrial enzymes in live cells. Subsequently, SDS in 0.01 M HCl was added to dissolve the cell membranes and formazan crystals during an overnight incubation at 37 °C. The level of conversion to formazan, as a relative measure of cell viability and mitochondrial metabolic activity, was documented by measuring optical density at 470 nm using a PowerWaveX microplate reader (BioTek Instruments). The data from PBMC viability assays were analyzed using the two-tailed *t*-tests.

## Results

### General Features of the Genome of Strain JBI-YZ6.3

**Fig. 1:**
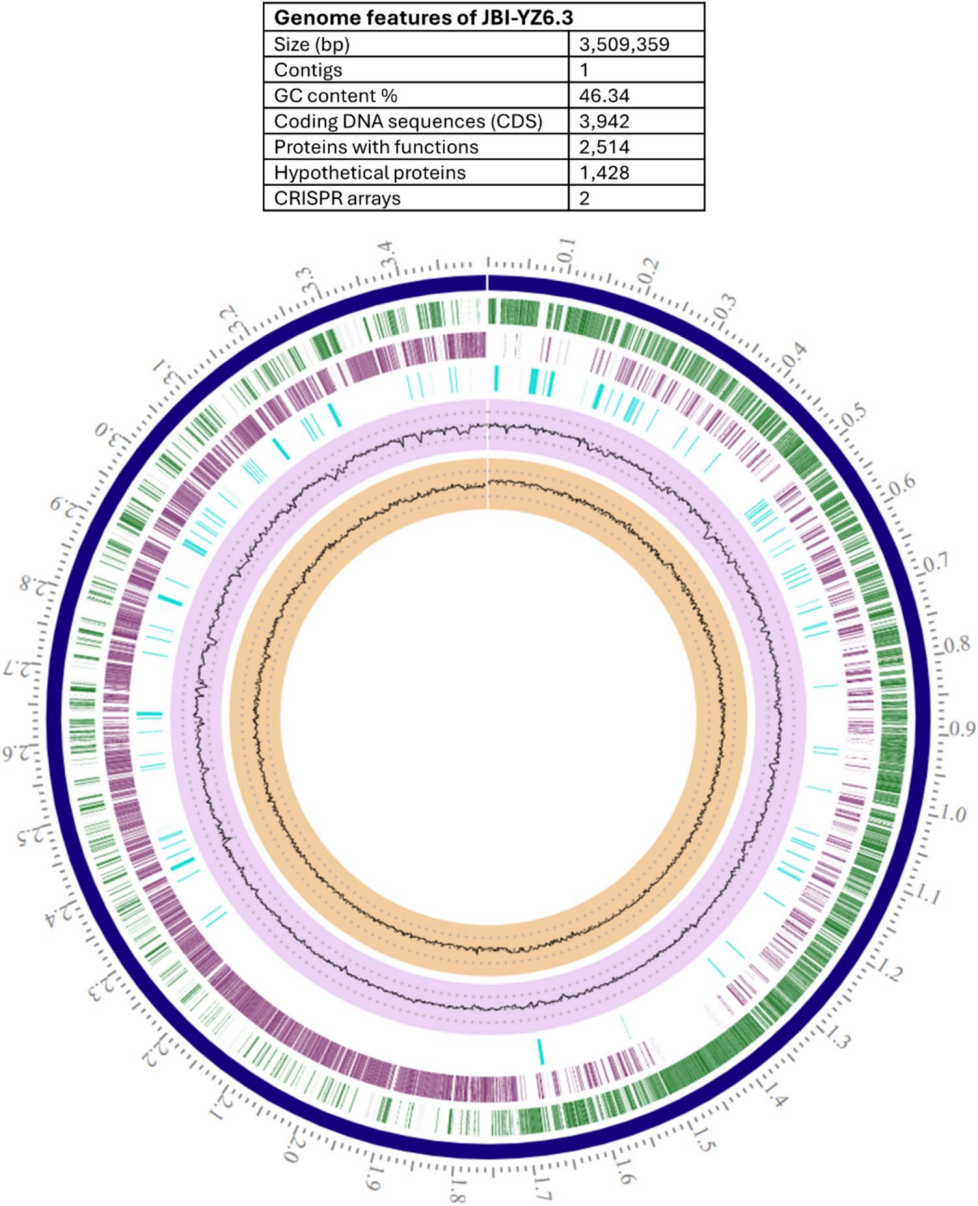
Circular genome map of *B. coagulans* strain JBI-YZ6.3. From outside to inside, circular genome shows position label (Mbp), contig/chromosome, CDS-forward strand, CDS-reverse strand, non-CDS features, GC content, and GC skew. The circular genome map of JBI-YZ6.3 was constructed using BV-BRC Annotation tool (https://www.bv-brc.org/app/Annotation)

Whole genome sequencing of strain JBI-YZ6.3 was carried out using the long-read PacBio platform [18]. The complete genome of *B. coagulans* JBI-YZ6.3 consists of one circular chromosome of 3.5 Mb with G + C content of 46.34% (Fig. 1). Genome annotation was performed using Rapid Annotation using Subsystem Technology (RAST), and 84 tRNAs, 30 rRNAs, and 2 CRISPR arrays were predicted in the genome. Among the 3942 coding sequences, 2514 are for proteins with functions, and the remaining 1428 are hypothetical proteins. No plasmid DNA was detected in the strain JBI-YZ6.3.

### Taxonomic Analysis of *B. coagulans* Strain JBI-YZ6.3

**Fig. 2:**
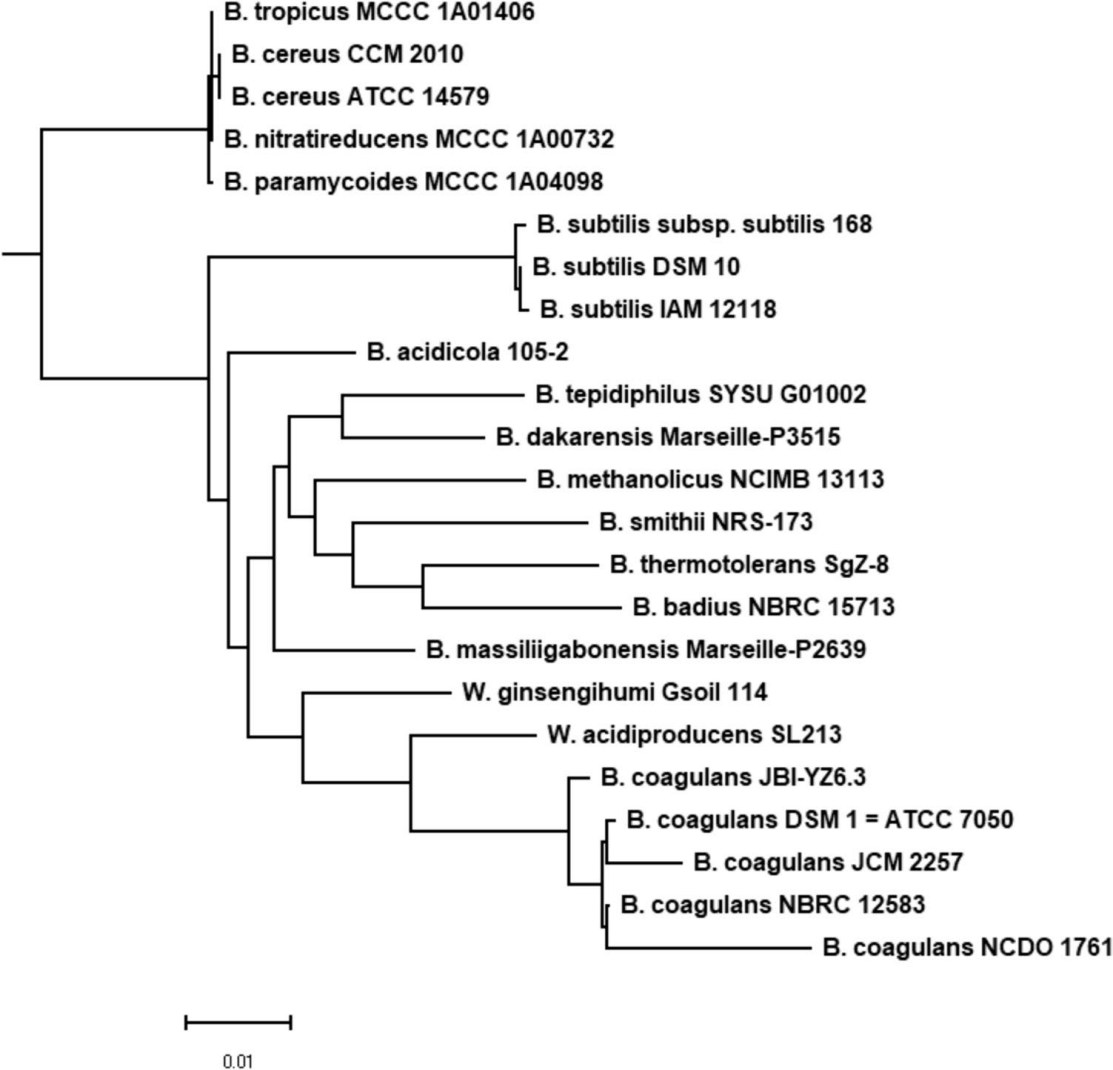
Phylogenetic tree based on 16S-rRNA of type strains of bacteria. FastME inferred from pairwise alignment between JBI-YZ6.3 16S-rRNA sequence from Sanger sequencing with type strain 16S-RNA sequences in the NCBI database

**Fig. 3:**
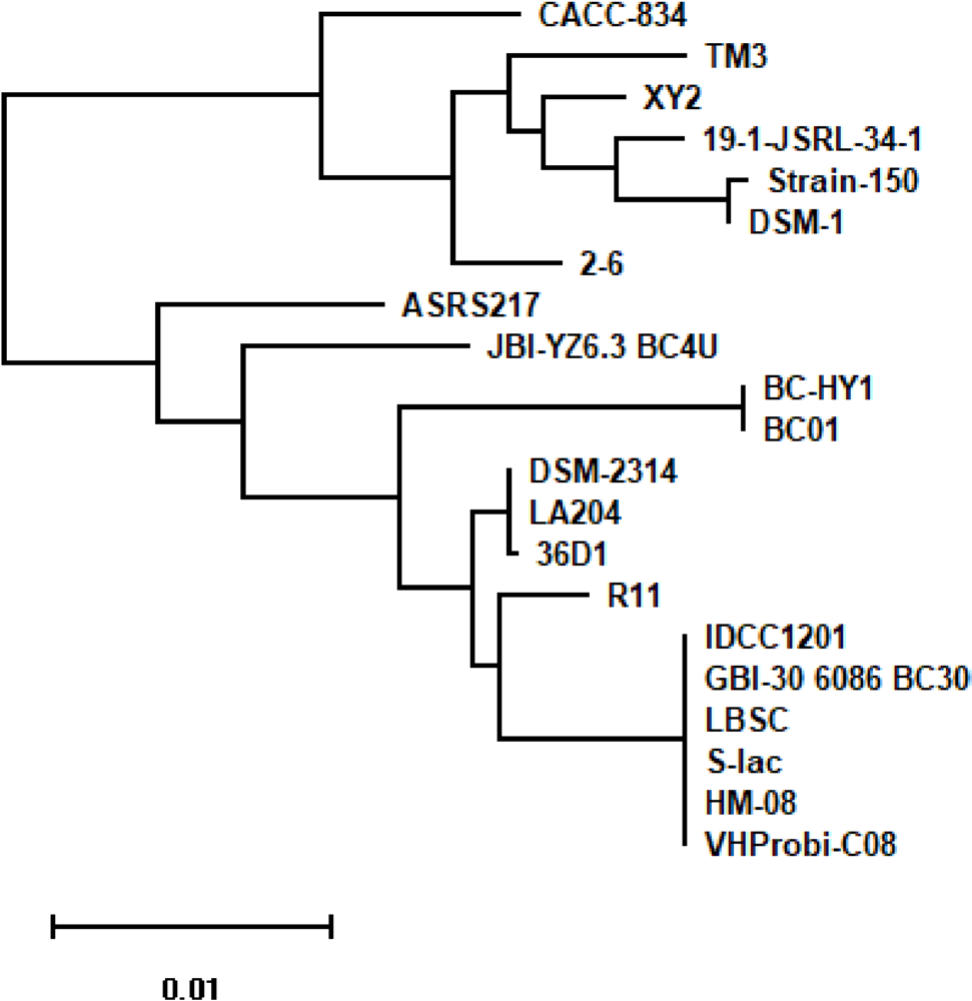
Phylogenetic tree based on MLST profiling. The five gene sequences from MLST analysis of strain JBI-YZ6.3 were concatenated and compared with homologous sequences from twenty *B. coagulans* strains. Phylogenetic distance inferred with the neighbor-joining clustering method (Clustal Omega v 1.2.4) was calculated and displayed using the Molecular Evolutionary Genetics Analysis (MEGA v. 10.0.4) program. The branch labels are the strain names of *B. coagulans*

The taxonomy of strain JBI-YZ6.3 was analyzed by 16S-rRNA and genome-based phylogeny, as well as analytical profile index (API) classification based on carbohydrate metabolism. Sequence similarity search using NCBI standard BLASTN showed that 16S-rRNA of strain JBI-YZ6.3 shared greater than 99% sequence identity to *B. coagulans* type strain ATCC 7050 with 100% coverage. The fast minimum evolution (FastME) phylogenetic tree based on the 16S-rRNA sequence identified strain JBI-YZ6.3 closely related to the *B. coagulans* (*W. coagulans*) type strains (Fig. 2). Fragments of five genes were used in the MLST analysis: DNA gyrase subunit B (*gyrB*), dihydroxy-acid dehydratase (*ilvD*), lactate dehydrogenase (*ldh*), phosphate acetyltransferase (*Pta*), and RNA polymerase β-subunit (*rpoB*). Multiple sequence alignment of the MLST sequences of strain JBI-YZ6.3 with twenty *B. coagulans* strains showed that JBI-YZ6.3 was a unique strain of *B. coagulans* (Fig. 3). The five gene fragments of strain JBI-YZ6.3 contained unique sequence variations compared with the other twenty strains and shared the least sequence identity with strain CACC-834 (95.63%) and the highest identity with strain 36D1 (98.44%). Genome-based phylogenetic tree, which was generated by the Codon Tree pipeline in the Bacterial and Viral Bioinformatics Resource Center (BV-BRC) [24] using 500 randomly selected genes, also demonstrated JBI-YZ6.3 as a unique strain among the group of *B. coagulans* strains used in the MLST analysis (Fig. S1).

The carbohydrate metabolic profile of strain JBI-YZ6.3 further supported the sequence-based taxonomic classification. The ability of strain JBI-YZ6.3 to metabolize carbohydrates was tested using the API 50 CH strip, which consists of a total of 50 different carbohydrates and derivatives (heterosides, polyalcohols, and uronic acids). Strain JBI-YZ6.3 was positive for various 5-carbon monosaccharides, 6-carbon monosaccharides, disaccharides, glycosides, and sugar alcohols (Table S2). The results were analyzed by APIWEB™, and strain JBI-YZ6.3 was identified as *Bacillus coagulans* with %ID of 99.8%. Interestingly, strain JBI-YZ6.3 was chiral specific for two pentose substrates: negative for D-arabinose, but positive for L-arabinose; positive for D-xylose, but negative for L-xylose. The chiral-specific metabolism of pentose is not a unique phenotype of strain JBI-YZ6.3 and is commonly found in bacteria. D-xylose and L-arabinose are the second and third most abundant sugars in plant lignocellulose biomass, respectively (D-glucose is most abundant) [25, 26]. Therefore, bacteria have evolved to metabolize these two sugars to support their growth. A recent report on another *B. coagulans* strain, CGI314, also showed the same activity [27].

### Comparison of JBI-YZ6.3 Genome with Published *B. coagulans* Genomes

The genome of JBI-YZ6.3 was compared with eight *B. coagulans* strains with complete genome sequences using three different genome-wide similarity comparison programs. The Similar Genome Finder searches for similar public genomes to the query genome in the BV-BRC database and computes genome distance estimation using Mash/MinHash. Strain DSM 2314 was identified as the most similar genome to JBI-YZ6.3 with the k-mer counts at 617/1000 (Table 1). Whole-genome distance based on digital DNA-DNA hybridization (DDH) of strain JBI-YZ6.3 with the eight known *B. coagulans* genomes was calculated using the Genome-Genome Distance Calculator (GGDC) 3.0 server. Consistent with the k-mer counts, JBI-YZ6.3 shared the highest similarity with DSM 2314 of 86.4% with the lowest distance among the strains in the comparison (Table 1). The average nucleotide identity (ANI) calculator estimates the distribution of nucleotide identity between fragments of two closely related genomes [28]. The genome of JBI-YZ6.3 showed the highest two-way ANI value to DSM 2314 at 98.67% (Table 1), above the threshold value of 95% for two genomes of the same species. The three genome-wide similarity programs ranked the eight *B. coagulans* genomes in the same order according to their similarities to strain JBI-YZ6.3 (Table 1).

**Table 1:** Comparison of JBI-YZ6.3 genome with other *B. coagulans* strains

Strain	GenBank sequence	Size (Mb)	GC%	Similar Genome Finder		DDH^b^		ANI^c^
				k-mer counts^a^	Distance	%	Distance	
JBI-YZ6.3	CP104390	3.51	46.34	1000/1000	0	100%	0	100
DSM 2314	CP033687	3.63	46.24	617/1000	0.012872	86.4	0.0160	98.67
LA-204	CP025437	3.64	46.18	614/1000	0.0130157	86.4	0.0161	98.63
S-lac	CP011939	3.69	46.23	608/1000	0.0133059	85.5	0.0170	98.53
LBSC (DSM 17654)	CP022701	3.64	46.28	608/1000	0.0133059	85.5	0.0171	98.51
HM-08	CP010525	3.63	46.28	607/1000	0.0133547	85.3	0.0172	98.51
R11	CP026649	3.60	46.34	594/1000	0.0139988	84.9	0.0177	98.47
2–6	CP002472	3.07	47.29	277/1000	0.0397669	62.4	0.0476	95.15
ATCC 7050	CP009709	3.37	46.90	245/1000	0.0444041	60.7	0.0503	94.87

^a^k-mer counts were calculated using the Similar Genome Finder tool of BV-BRC (https://www.bv-brc.org/app/GenomeDistance)

^b^DDH with GGDC 3.0 Formula 2 (https://ggdc.dsmz.de/ggdc.php)

^c^ANI (Average Nucleotide Identity) was calculated using the ANI Calculator (http://enve-omics.ce.gatech.edu/ani/)

### Shelf-Life Stability

**Fig. 4:**
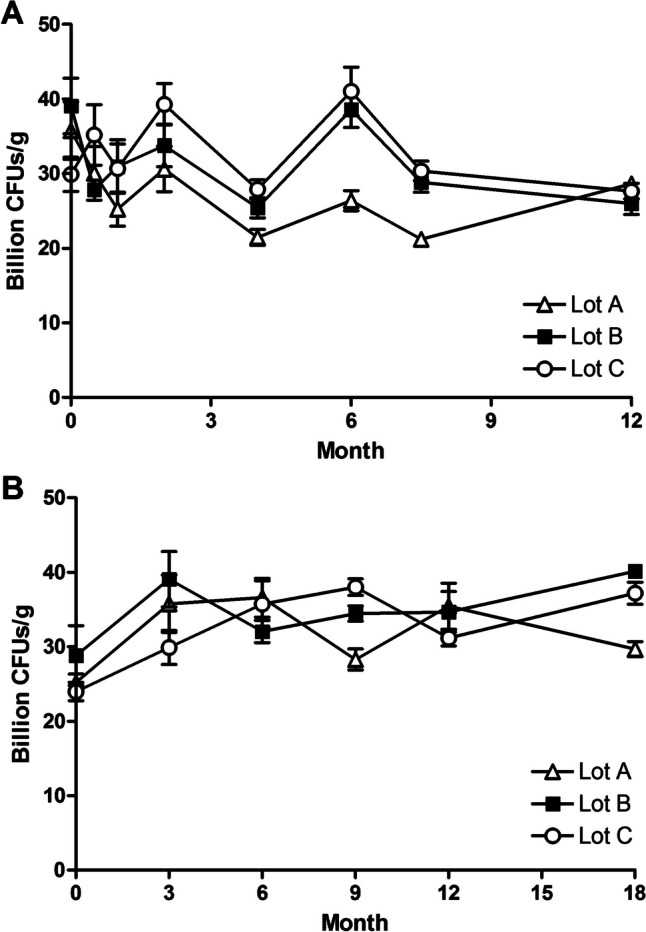
Viability of JBI-YZ6.3 spores stored at **A** 40 °C and 75% relative humidity, **B** room temperature (20–25 °C and ambient relative humidity (30–60%). Data are shown as means ± SEM from three independent batches (Lot A, B, and C) of JBI-YZ6.3 dry spores

The stability of JBI-YZ6.3 was monitored under two different conditions of temperature and relative humidity (RH): accelerated test at 40 °C and 75% RH for 12 months and long-term test at room temperature and ambient RH for 18 months [29]. Spores of JBI-YZ6.3 were blended with maltodextrin to reach no less than 15 billion CFU/gram. Three different production lots were used as a representative sample group and stored in heat-sealed aluminum bags. Samples from each of the production lots were collected and analyzed at different time intervals. No significant loss of total viable spore counts was observed in the accelerated test up to the termination point of 12 months, as well as in the long-term test up to the termination point of 18 months (Fig. 4). No changes in the product’s color and texture were noted during the testing periods for both conditions. Spores of strain JBI-YZ6.3 without blending with maltodextrin showed a similar stability trend to the blended spores. These results demonstrated that JBI-YZ6.3 spores are stable for long-term storage.

### Acid and Bile Tolerance

**Fig. 5:**
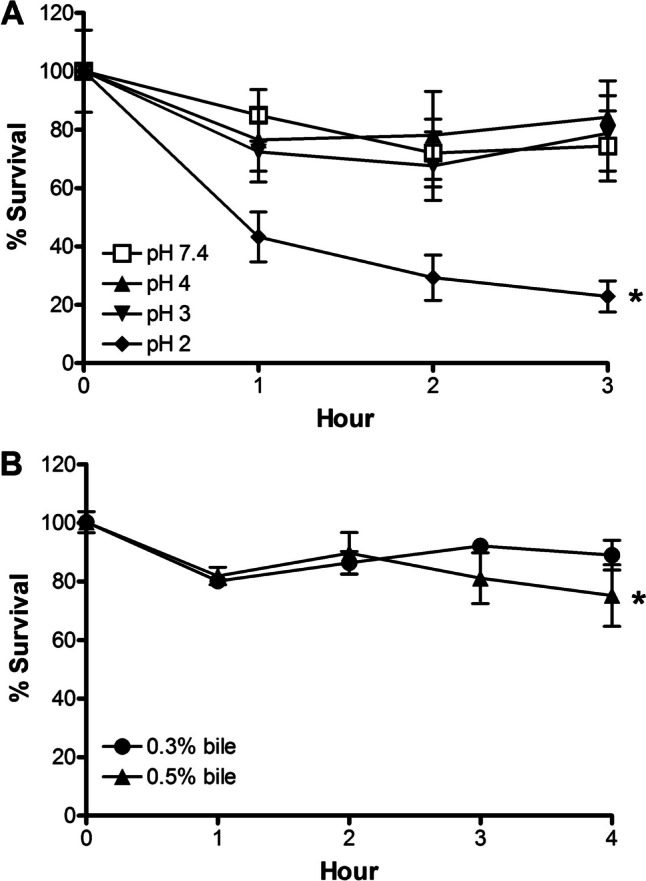
Survival of JBI-YZ6.3 spores at different **A** acidic pH and **B** bile concentrations. Data are shown as means ± SEM, and comparisons among survival at different pH and bile concentrations were made by 2-way ANOVA. Comparison with the control treatment with *p* value < 0.05 was considered statistically significant and indicated by an asterisk mark (*)

Probiotics must be able to survive the passage through the stomach and small intestine to extend benefits to the hosts. Therefore, resistance to the acidic gastric juice, ranging between pH 1.5 and 3.5, and the bile salts in the small intestine is required. Spores of JBI-YZ6.3 were challenged under acidic conditions (pH 4, 3, and 2) for 1-h increments over 3 h, and their survival was measured and compared to a control (pH 7.4). Spores challenged at pH 3 and 4 showed no significant decrease in viability, and their survival curves were indistinguishable from the control (Fig. 5A). However, the spore viability was reduced when challenged at pH 2, with approximately 25% survival after 3 h. Similarly, JBI-YZ6.3 spores were challenged with 0.3% and 0.5% bile for up to 4 h, and their survival was determined at 1 h intervals (Fig. 5B). The survival rate of JBI-YZ6.3 after 4 h in the presence of 0.3% and 0.5% bile was 90% and 75%, respectively. These results demonstrated that JBI-YZ6.3 can tolerate harsh conditions during the passage through the stomach and small intestine.

### Antibiotic Susceptibility

The susceptibility of strain JBI-YZ6.3 to a panel of antibiotics including the eight antibiotics recommended by EFSA was tested [30]. The MIC values ranged between 0.016 and 2 µg/mL and were below the EFSA breakpoints for sensitive phenotype (Table 2). In addition, zones of inhibition (ZOI) using antibiotic discs were also tested. The diameters of the clearing zones were larger than the 20 mm breakpoint value for susceptible organisms defined by the Clinical and Laboratory Standards Institute (Table S3). The genomic sequence of JBI-YZ6.3 was evaluated for antimicrobial resistance genes using two genome-wide screening programs, ResFinder and the Comprehensive Antibiotic Resistance Database (CARD). Consistent with the sensitive phenotypes observed from the antibiotic susceptibility tests, no potential antibiotic resistance genes were found in this strain.

**Table 2:** The MIC values of *B. coagulans* strain JBI-YZ6.3 to antibiotics

**Antibiotic**	**MIC (μg/ml)**	**EFSA Breakpoint for** ***Bacillus*** **spp.** **(μg/mL)**	**Interpretation**
Gentamicin	0.016	4	S
Kanamycin	0.19	8	S
Streptomycin	0.25	8	S
Tetracycline	0.032	8	S
Erythromycin	0.38	4	S
Clindamycin	0.125	4	S
Chloramphenicol	2	8	S
Vancomycin	0.38	4	S
Ampicillin	0.5	Not required	S
Rifampicin	0.023	Not required	S
Linezolid	0.75	Not required	S
Ciprofloxacin	0.25	Not required	S
Quinupristin-dalfopristin	0.125	Not required	S

*S* sensitive

### Detection of Enterotoxins and Cytotoxicity

The genome of JBI-YZ6.3 was screened for any potential toxigenic genes using known *Bacillus* toxin genes including enterotoxins as queries. No matches for toxin genes were identified using BLASTN searches from a panel of 13 *Bacillus* toxin genes (Table S4). *Bacillus cereus* enterotoxin-reversed passive latex agglutination (BCET-RPLA) kit detects the L2 component of hemolysin BL [31]. In vitro testing using the BCET-RPLA kit showed no detectable level of the toxin in the culture supernatant of JBI-YZ6.3. Cytotoxicity of JBI-YZ6.3 was tested in vitro against Vero epithelial cells using lactate dehydrogenase (LDH) release assay in accordance with EFSA’s guidance [22]. The maximum LDH release was established using a lysis solution (100%). Vero cells treated with JBI-YZ6.3 culture supernatant at 10% concentration released a low level of LDH at 1.9%, well below the 20% threshold indicative of cytotoxicity.

### PBMC Viability and Mitochondria Metabolic Activity in the Presence of JBI-YZ6.3

**Fig. 6:**
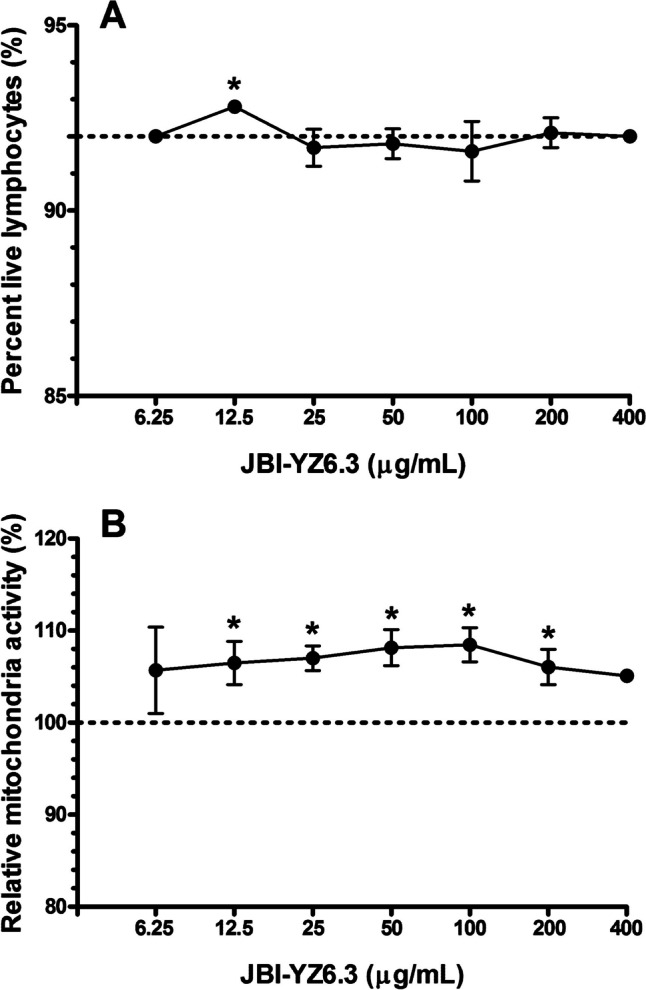
The effects of strain JBI-YZ6.3 on the viability of human peripheral blood mononuclear cells (PBMC). The viability of PBMC was determined by flow cytometry **A** and mitochondria activity by the MTT assay **B**. Data are shown as means ± SD from three independent experiments. Comparisons between JBI-YZ6.3-treated and control PBMC analyzed using the two-tailed unpaired *t*-test. Statistical significance was set at *p* value < 0.01 and indicated by an asterisk (*)

The viability of human PBMC in the presence of germinated spores of JBI-YZ6.3 was determined by flow cytometry. After co-incubation of PBMC with JBI-YZ6.3 for 20 h, no reduction of cell viability was detected in PBMC in the presence of JBI-YZ6.3 compared with the untreated control (Fig. 6A). Cellular energy production measured by the MTT assay is an indicator of relative mitochondrial metabolic activity. PBMC cultures in the presence of JBI-YZ6.3 exhibited no reduction of mitochondrial metabolic activities (Fig. 6B). In contrast, JBI-YZ6.3 treatment showed significantly higher mitochondrial activities in five concentrations tested compared with the control (Fig. 6B).

## Discussion

The safety of *Bacillus coagulans* JBI-YZ6.3 as a new probiotic strain was critically evaluated using genomic analyses and phenotypic-based analyses. Because regulatory authorities such as EFSA and FDA apply safety guidelines and standards on bacterial probiotic strains based on taxonomic classification [22, 30], it is critical to precisely determine a new strain’s taxonomy before considering its safety and probiotic efficacy. We established unequivocally that strain JBI-YZ6.3 is *B. coagulans* using 16S-rRNA and genome-based phylogenetic analyses, in addition to biochemical testing using API. Strain JBI-YZ6.3 demonstrated tolerance to acid and bile salts, as well as stability in long-term shelf-life studies. No antibiotic resistance was detected in strain JBI-YZ6.3 by both antibiotic susceptibility testing and in silico search for antibiotic resistance genes. Strain JBI-YZ6.3 showed no cytotoxicity towards Vero cells and human PBMC.

*B. coagulans* has gained growing interest in probiotic development due to its combined advantages of being a spore-former and lactic-acid producer [32–34]. The NCBI bacterial genome database contains 61 *B. coagulans* genome assemblies as of August 2023. Comparison of the genome of JBI-YZ6.3 with other *B. coagulans* strains demonstrated that strain JBI-YZ6.3 is a unique new strain (Figs. 3 and S1, Table 1). Interestingly, six strains of *B. coagulans*, including IDCC1201, GBI-30 6086, LBSC, S-lac, HM-08, and VHProbi-C08, are identical in the MLST analysis using five conserved gene fragments (Fig. 3). These strains were also clustered together in the genome-based phylogenetic tree (Fig. S1). Further genomic sequence mining could uncover new functions/activities of strain JBI-YZ6.3.

*Bacillus* species other than the *B. cereus* group organisms rarely cause foodborne diseases [35]. However, various regulatory agencies require potential *Bacillus* probiotic strains to be tested for enterotoxins and cytotoxicity. The cytotoxicity of strain JBI-YZ6.3 was tested using different methods towards both Vero epithelial cells and human PBMC. No toxicity was observed, and surprisingly, the human PBMC showed increased mitochondrial activity in the presence of JBI-YZ6.3 (Fig. 6B). This could be due to the antioxidative effect of *B. coagulans*, which has been reported previously [36]. Further studies are warranted to determine if strain JBI-YZ6.3 has protective effects on human and animal cells due to antioxidative activities.

Probiotic bacteria are a good source of essential nutrients for the host and the gut microbiota. The genome of JBI-YZ6.3 contains over 100 genes with annotated functions in the biosynthesis of B-group vitamins (Table S5), including thiamine (B1), riboflavin (B2), nicotinamide (B3), pantothenate (B5), pyridoxin (B6), biotin (B7), and folate (B9). Strain JBI-YZ6.3 contains 78 genes for the biosynthesis of essential amino acids, and three genes that encode cholesterol-lowering enzymes/proteins were also predicted. Additionally, functions that promote JBI-YZ6.3 adhesion, retention, and survival in the GI tract were predicted, including proteins involved in adherence to the gut epithelium (fibronectin/fibrinogen-binding protein and flagellar hook-associated proteins FlgK and FlgL) and stress adaptation proteins (chaperone proteins, Clp protease, and heat- and cold-shock proteins). Furthermore, strain JBI-YZ6.3 has 71 genes with annotated functions associated with sporulation. PHASTER identified four prophage regions in the genome of JBI-YZ6.3, but none of them was predicted to be a complete prophage. BAGEL4 was used to search the genome of JBI-YZ6.3 for potential bacteriocin gene clusters. Similar to strain PL-W, two areas of interest were identified: one encoded a circularin A and the other an amylocyclicin [37].

iProbiotics program is a machine-learning-based platform for rapid identification of probiotic properties using a collection of 184 core features [38]. The program has a high degree of prediction accuracy (97.77%). Using the whole genome sequence of JBI-YZ6.3, the iProbiotics program predicted the probability of JBI-YZ6.3 for probiotics was 0.9943, and the probability for non-probiotics was 0.0057.

In conclusion, strain JBI-YZ6.3 is a unique new probiotic strain of *Bacillus coagulans* that showed a good safety profile demonstrated by the lack of antibiotic resistance and cytotoxicity, exhibited good ability to survive in the gut (low pH and bile), and showed good long-term stability. These features provide the basis for strain JBI-YZ6.3 to be further studied and developed for its probiotic activities.

## Supplementary Information

Below is the link to the electronic supplementary material.

Supplementary file1 (DOCX 2177 KB)

## Data Availability

All data are included in the text; however, the raw data of this article will be made available by the authors, without undue reservation, to any qualified researcher.
