# Supplementary material for: Genomic and Phenotypic Safety Assessment of Probiotic Bacillus coagulans Strain JBI-YZ6.3
Source: Probiotics Antimicrob Proteins. 2024 Jun 19;17(5):3440–50. doi: 10.1007/s12602-024-10305-4 (PMC12532747; doi:10.1007/s12602-024-10305-4)
Supplement: Supplementary file 1 — Supplementary file1 (DOCX 2177 KB) [file 12602_2024_10305_MOESM1_ESM.docx]

Table S1. Primers used in MLST analysis.

| **Product** | **Forward Primer** | **Reverse Primer** | **Size (bp)** |
| --- | --- | --- | --- |
| gyrB | AACTTGCAGACTGCACATCACG | AATTCCGCCGCGGTTCCAC | 645 |
| ilvD | GCGGTTCAACCAATACCG | ATATCTTGCCAGGTAGCCG | 824 |
| ldh | GGAAGTCATGGCAGGGATTA | CCYGAATATACGATTTTCCGG | 659 |
| pta | GTGAGTACATTTTTTGAYGG | CCACTTTCTCCGTCTCATC | 652 |
| rpoB | GAACTTGCCCTTGGCCAGAA | CCGGCTTCTTCAATCGTTTC | 770 |

Table S2. Carbohydrate metabolism test results of *B. coagulans* strain JBI-YZ6.3 using API 50 CHB

| Tube # | Substrate | JBI-YZ6.3 |
| --- | --- | --- |
| 0 | Control | – |
| 1 | Glycerol | – |
| 2 | Erythritol | – |
| 3 | D-Arabinose | – |
| 4 | L-Arabinose | + |
| 5 | D-Ribose | + |
| 6 | D-XyLose | + |
| 7 | L-XyLose | – |
| 8 | D-Adonitol | – |
| 9 | Methyl-βD-Xylopyranoside | – |
| 10 | D-Galactose | + |
| 11 | D-Glucose | + |
| 12 | D-Fructose | + |
| 13 | D-Mannose | + |
| 14 | L-Sorbose | – |
| 15 | L-Rhamnose | + |
| 16 | Dulcitol | – |
| 17 | Inositol | – |
| 18 | D-Mannitol | – |
| 19 | D-Sorbitol | + |
| 20 | Methyl-αD-Mannopyranoside | – |
| 21 | Methyl-αD-Glucopyranoside | – |
| 22 | N-Acetylglucosamine | + |
| 23 | Amygdalin | + |
| 24 | Arbutin | + |

| Tube # | Substrate | JBI-YZ6.3 |
| --- | --- | --- |
| 25 | Esculin | + |
| 26 | Salicin | + |
| 27 | D-Cellobiose | + |
| 28 | D-Maltose | + |
| 29 | D-Lactose (bovine origin) | – |
| 30 | D-Melibiose | + |
| 31 | D-Saccharose (sucrose) | – |
| 32 | D-Trehalose | + |
| 33 | Inulin | – |
| 34 | D-Melezitose | – |
| 35 | D-Raffinose | – |
| 36 | Amidon (starch) | – |
| 37 | Glycogen | – |
| 38 | Xylitol | – |
| 39 | Gentiobiose | + |
| 40 | D-Turanose | – |
| 41 | D-Lyxose | – |
| 42 | D-Tagatose | – |
| 43 | D-Fucose | – |
| 44 | L-Fucose | – |
| 45 | D-Arabitol | – |
| 46 | L-Arabitol | – |
| 47 | Potassium Gluconate | + |
| 48 | Potassium 2-Ketogluconate | – |
| 49 | Potassium 5-Ketogluconate | – |

Table S3. The susceptibility of *B. coagulans* strain JBI-YZ6.3 to antibiotics by zone of inhibition (ZOI)

| **Antibiotic** | **ZOI (mm)** | **Interpretation** |
| --- | --- | --- |
| Gentamicin | 23 | S |
| Kanamycin | 26 | S |
| Streptomycin | 27 | S |
| Tetracylcine | 39 | S |
| Erythromycin | 33 | S |
| Clindamycin | 30 | S |
| Chloramphenicol | 35 | S |
| Vancomycin | 25 | S |
| Ampicillin | 23 | S |
| Rifampcin | 34 | S |

S: sensitive based on the Clinical and Laboratory Standards Institute (CLSI) breakpoint/interpretive criteria (Clinical and Laboratory Standards Institute, 2012).

Table S4. Summary of BLASTN search results for *Bacillus* toxin genes in JBI-YZ6.3

| **Gene** | **Organism** | **Gene ID** | **Match** |
| --- | --- | --- | --- |
| Hemolysin BL (*hbl*) | | | |
| *hblA* | *B. cereus* strain 5.39 | KF681259.1 | No match |
| *hblC* | *B. cereus* strain EC230 | JQ039142.1 | No match |
| *hblD* | *B. cereus* strain EC303 | JQ039158.1 | No match |
| *hblD* | *B. cereus* ATCC 14579 | 50193893 | No match |
| Non-hemolytic enterotoxin (*nhe*) | | | |
| *nheABC* | *B. cereus* strain NVH 391/98 | DQ885236.1 | No match |
| *nheABC* | *B. weihenstephanensis* strain WSBC 10386 | DQ153260.1 | No match |
| *nheABC* | *B. cereus* strain 1230/88 | Y-19500.2 | No match |
| *nhe* locus | *B. thuringiensis* serovar kurstaki strain VBTS 2477 | EU925144.1 | No match |
| Cytotoxin K (*cytK*) | | | |
| *cytK* | *B. cereus* strain NVH0391/98 | AJ277962.1 | No match |
| *cytK* | *B. cereus* strain F4430/73 | DQ019311.1 | No match |
| *cytK* | *B. mycoides* strain BFDA 5521 | AY871809.1 | No match |
| Cereulide (*ces*) | | | |
| *cesA* | *B. cereus* strain F4810/72 | ABK00751.1 | No match |
| *cesB* | *B. cereus* strain F4810/72 | ABK00633.1 | No match |

Table S5. Genome features of JBI-YZ6.3 related to beneficial probiotic activities

| **Probiotic functions/activities** | **Gene / Feature counts** |
| --- | --- |
| ***Vitamin biosynthesis*** | ***104*** |
| Thiamine (B1) | 12 |
| Riboflavin (B2) | 15 |
| Nicotinamide (B3) | 10 |
| Pantothenate (B5) | 4 |
| Pyridoxin (B6) | 1 |
| Biotin (B7) | 19 |
| Folate (B9) | 43 |
| ***Cholesterol lowing enzymes/proteins*** | ***3*** |
| ***Adhesion*** | ***2*** |
| ***Essential amino acid biosynthesis*** | ***78*** |
| Threonine | 13 |
| Tryptophan | 10 |
| Methionine | 27 |
| Leucine | 6 |
| Lysine | 10 |
| Phenalanine | 6 |
| Histadine | 6 |
| ***General stress adaptation proteins*** | ***9*** |
| Chaperone protein | 4 |
| Cold shock protein | 4 |
| Heat shock protein | 1 |
| ***Sporulation*** | ***71*** |


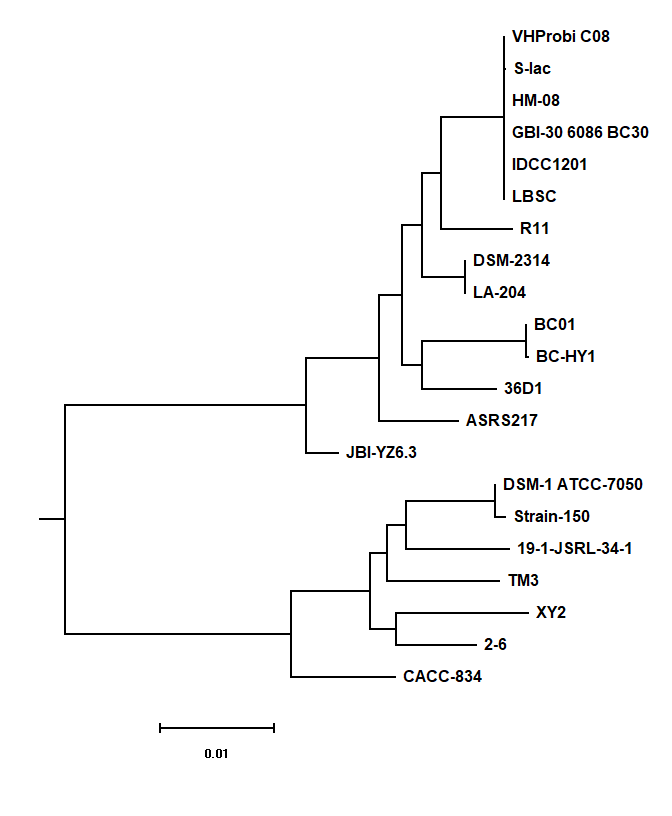


Figure S1. Genome-based phylogenetic tree of twenty *B. coagulans* strains. The BV-BRC Codon Tree (<https://www.bv-brc.org/app/PhylogeneticTree>) pipeline randomly selected 500 genes, built an alignment, and generated a tree based on the differences within the selected sequences.
